# Whole-transcriptome changes in gene expression accompany aging of sensory neurons in *Aplysia californica*

**DOI:** 10.1186/s12864-018-4909-1

**Published:** 2018-07-11

**Authors:** Justin B. Greer, Michael C. Schmale, Lynne A. Fieber

**Affiliations:** Department of Marine Biology and Ecology, Rosenstiel School of Marine and Atmospheric Science, University of Miami, 4600 Rickenbacker Cswy, Miami, FL 33149 USA

**Keywords:** Transcriptomics, Pleural ganglion, NMDA

## Abstract

**Background:**

Large-scale molecular changes occur during aging and have many downstream consequences on whole-organism function, such as motor function, learning, and memory. The marine mollusk *Aplysia californica* can be used to study transcriptional changes that occur with age in identified neurons of the brain, because its simplified nervous system allows for more direct correlations between molecular changes, physiological changes, and their phenotypic outcomes. Behavioral deficits in the tail-withdrawal reflex of aged animals have been correlated with reduced excitation in sensory neurons that control the reflex. RNASeq was used to investigate whole-transcriptome changes in tail-withdrawal sensory neurons of sexually mature and aged Aplysia to correlate transcriptional changes with reduced behavioral and physiological responses.

**Results:**

Paired-end sequencing resulted in 210 million reads used for differential expression analysis. Aging significantly altered expression of 1202 transcripts in sensory neurons underlying the tail-withdrawal reflex, with an approximately equal number of these genes up- and down regulated with age. Despite overall bidirectionality of expression changes, > 80% of ion channel genes that were differentially expressed had decreased expression with age. In particular, several voltage-gated K^+^ and Ca^2+^ channels were down regulated. This marked decrease in ion channel expression may play an important role in previously observed declines in aged sensory neuron excitability. We also observed decreased expression of genes and pathways involved in learning and memory. Genes involved in the stress response showed increased expression in aged Aplysia neurons.

**Conclusions:**

Significantly altered expression of many genes between sexually mature and aged Aplysia suggests large molecular changes that may impact neuronal function. Decreased ion channel mRNA observed could mean fewer receptors present in aged neurons, resulting in reduced excitability of PVC sensory neurons, ultimately leading to reduced tail-withdrawal reflex observed in aged Aplysia. Significant changes in other genes and pathways, such as stress response and learning and memory, have previously been shown to occur with age in many vertebrate organisms. This suggests that some effects of aging are common across many animal phyla.

**Electronic supplementary material:**

The online version of this article (10.1186/s12864-018-4909-1) contains supplementary material, which is available to authorized users.

## Background

Aging changes at the cellular and molecular levels result in declines in learning and memory, locomotion, and reflex systems [1–4]. *Aplysia californica* (Aplysia), the California sea hare, is a marine mollusk that has been used extensively in research correlating physiological and behavioral declines with gene expression changes in neuronal circuits during aging [4–10], due to a less complex nervous system than vertebrates. Changes in behavior with age can be studied at the physiological and molecular level in individual neurons that control the behaviors. Additionally, an annual lifespan allows these studies to be completed in a relatively short period of time.

Previous studies in Aplysia have shown that reflex impairments in aged animals corresponded to declines in neuronal excitability in the neurons that underlie the behavior [4–7, 11]. Physiological aging, such as reduced motoneuron excitability, has also been correlated with transcriptional changes such as reduced expression of membrane receptors [11] and changes in cellular pathways that ensure normal function [13]. For example, alterations in acetylcholine-induced action potentials in the R15 neuron during aging occurred in conjunction with reduced expression of some acetylcholine receptors [11]. Biological pathways for cell signaling, cellular function, and neurological diseases are also altered in aged R15 neurons in Aplysia [12].

Aging in sensory neurons is understudied, despite their role in responding to external stimuli to coordinate voluntary movements, some of which show stereotyped changes in aging animals [13]. To better characterize sensory neuron aging in Aplysia, we identified molecular changes with age in the pleural ventral caudal (PVC) sensory neurons located in the pleural ganglia. PVC sensory neurons are of particular interest due to their involvement in the tail-withdrawal reflex (TWR). Direct tail stimulation initiating TWR activates PVC sensory neurons, which make monosynaptic connections to three motoneurons of the pedal ganglion (P7-P9) and trigger movement of the tail muscle [14].

Aplysia can be classified as Mature (M, ~ 8 months) or Aged II (AII, ~ 12 months) based on diminished TWR behavior, as well as physiological changes in the underlying neural circuits. Predictable increases in TWR time occur in AII compared to M [5], and these declines are associated with reduced excitability in both PVC sensory neurons and pedal motoneurons underlying TWR, as well as reduced conduction velocity [4, 5, 15]. Our laboratory has also described declines in glutamatergic neurotransmission in sensory and motoneurons of TWR with age [4, 5]. Reduced excitability of each of the constituents of the TWR suggest synaptic transmission is negatively affected by age, and contributes to increased reflex completion times in AII animals.

The cause of altered PVC function is a critical question that can provide insights into aging of TWR, and sensory neuron aging in general. In this study, we characterized the molecular correlates of altered PVC function in aged animals using RNASeq. First, behavioral experiments were conducted in mature and aged animals to confirm aging of TWR. Then, whole-transcriptome changes in PVC sensory neurons were assessed using the same animals assessed for reflex behaviors.

## Methods

### Animal rearing and behavioral assessments of aging

Two cohorts of Aplysia that originated from differently parented egg masses of wild-caught animals were reared at the University of Miami National Resource for Aplysia. Both cohorts were maintained at a maximum of 5 per cage and fed an ad lib diet of *Agardhiella subulata* throughout their life as previously described [16].

Sexual maturity (M) occurred at 8 months in both cohorts, and Aged II (AII) occurred at 12 months. Behavioral assessments were executed beginning at M, defined as the day the first egg mass was found, on 6–8 randomly selected animals from each cohort. Assessments were continued monthly on randomly selected animals from each cohort through the end of life, age ~ 12.5 months in both cohorts. Each monthly measurement of a reflex consisted of 3 repetitions on each animal, with a minimum of 5 min between trials.

Behavioral assessments were carried out using previously described protocols [5]. Briefly, TWR was initiated by pressing a blunted 21-gauge needle to the tip of the animal’s tail by approximately 1/2 the tail thickness for approximately 1 s to initiate withdrawal of the tail. Time was recorded from the initiation of tail withdrawal until the animal had relaxed the tail approximately 30%.

The righting reflex also exhibits declines in performance with age, and was chosen as a second measure of aging reflexes. Righting was recorded by picking an animal up and releasing it by tilting the palm of the hand at the top of the water column. Time for the reflex was recorded starting when the animal reached the cage bottom and ending when it took its first crawling step after righting itself, and adhered to the bottom of the cage.

### RNA extraction and sequencing preparation

RNA was extracted from PVC neurons from six animals from each cohort at M and six animals from each cohort at AII. The animals chosen for dissection of ganglia and RNA extraction from neural clusters were a randomly selected subset of the animals tested for TWR and righting at M and AII. This allowed for direct correlation of an individual animals’ behavioral assessments with its expression profile.

To remove PVC neurons for RNA extraction, animals were first anesthetized by injection of 1/6th body weight of isotonic MgCl_2_. The central nervous system (CNS) was then removed by severing the connectives and removal of all nervous system ganglia. PVC neurons were separated from the remainder of the pleural ganglion by pinning the pedal and pleural ganglia tightly in a sylguarded 35 mm plastic dish filled with artificial sea water (417 mM NaCl, 10 mM KCl, 10 mM CaCl2, 55 mM MgCl2, and 15 mM HEPES-NaOH, pH 7.6). PVC neurons were peeled away from the remainder of the pleural ganglion with a pair of fine forceps after removal of the surrounding connective tissue. PVC neurons from each hemiganglion were pooled as a single sample.

Total RNA was extracted with the Qiagen RNeasy Micro Kit (Cat. #74004) following the manufacturer’s instructions. Samples were treated with DNase to remove any contaminating DNA. RNA quantity and quality were assessed with both a Nanodrop (model ND-1000) and an Agilent 2100 Bioanalyzer prior to library preparation.

Two hundred ng of RNA from the 3 highest quality RNA samples from each cohort at M and AII were used in downstream library preparation. The highest quality RNA samples were defined as having sufficient RNA quantity and 260/280 nm ratio ~ 2 to ensure no DNA or protein contamination. Libraries were prepared using the Illumina TruSeq Stranded Total RNA Low-Throughput Library Prep Kit (Cat. #RS-122-2201) following the manufacturer’s instructions. Following library preparation, samples were assessed on the bioanalyzer for quantification and to verify 200-300 bp fragment sizes. Libraries were sequenced as 100 bp paired-end reads by Elim Biopharmaceuticals (Hayward, CA) in one lane on a HiSeq 2500 high-throughput sequencer (Illumina).

### Data processing

Raw reads were quality filtered using the fastaxtoolkit (<http://hannonlab.cshl.edu/fastx_toolkit/>). When a base pair quality score of < 20 was encountered in a read, the remainder of the read was cut off and removed. Reads shorter than 20 base pairs after trimming and removal of barcodes were also removed. Quality score and nucleotide distributions for each library were visualized using the Galaxy web server [17].

rRNA was removed during sequencing preparation, however some samples still contained rRNA reads in their libraries. In order to assess an accurate number of total reads used for downstream analysis, rRNA reads were removed from all samples prior to analysis by mapping all reads to the Aplysia rRNA annotation with the STAR aligner (parameters described below) and discarding reads that aligned to the rRNA annotation. Therefore, downstream analysis did not contain rRNA reads.

### Mapping reads to the genome and differential expression analysis

Aplysia exon information (ref_AplCal3.0_gnomon_scaffolds.gff3) and genome annotation (version AplCal3.0 from July, 2015), from the NCBI ftp server database, were used for genomic mapping. After quality filtering, reads were mapped to the Aplysia genome using the STAR aligner. Genome index files for use with STAR were generated using the --runMode genomeGenerate option using both the Aplysia AplCal3.0 genome and the ref_AplCal3.0_gnomon_scaffolds.gff3 transcript annotation. Quality controlled reads were aligned using the STAR aligner [18]. The --quantMode Genecounts option was utilized to count the number of reads uniquely mapping to each transcript using the HTSeq-count program [19]. Mapping and counting for paired-end and singletons were executed separately, and total counts for each transcript was determined by summing the counts for the paired and singletons reads for each sample.

Statistical testing for differential expression (DE) of transcript counts was performed using DeSeq2 [20], a method based on the negative binomial distribution and performed in the R statistical environment [21]. Only reads that uniquely mapped to the genome were counted and used for analysis. Raw read counts were normalized in DeSeq2 to adjust for differences in library sizes. Significant DE was defined as adjusted *p*-values≤0.05 (padj) after false discovery rate correction. Data were normalized using the regularized log transformation for principal component analysis (PCA).

Of the top 50 most significantly DE genes, 17 were classified as uncharacterized proteins. Each uncharacterized protein was analyzed in a BLAST search against human proteins and for conserved domains in InterPro [22] to attempt to annotate these genes. Three genes were annotated in this way: CCAP1, DNA ligase-like, and glycine N-acyltransferase-like. None of these three genes were previously annotated in the Aplysia transcriptome database.

### Gene ontology and canonical pathway analysis

Transcripts found to be DE were first translated into their corresponding protein sequences prior to gene ontology analysis. Translated sequences were BLASTed against the *Homo sapiens* refseq protein database (version 9606.9558) using Blast2GO (ver. 3.3, E-value≤1.0E-3) to identify proteins with human homologs for gene ontology (GO) analysis [23]. This allowed for mapping of DE genes to the better-studied and annotated human GO pathways. For each gene, GO annotation terms with E-value≤1.0E-6 were used in downstream enrichment analysis. This process was repeated for the full Aplysia transcriptome as a reference set for enrichment analysis.

GO terms of protein translations from DE transcripts were tested against the reference set of GO terms for the full Aplysia genome annotation to test for biological processes and molecular functions that were over-represented (enriched) in the DE proteins. Using a Fisher’s exact test, GO terms that were over-represented at a padj≤0.05 were identified.

Aplysia proteins with human homologs identified in Blast2GO were further analyzed using the Ingenuity Pathways Analysis (IPA) tool (Qiagen). Functional analysis in IPA was conducted using the core analysis feature to identify the pathways most affected during aging. For the confidence parameter, only experimentally observed relationships were used. This analysis also calculated directionality from log-fold changes to identify pathways that are up or down regulated via a z-score.

### qPCR validation of selected genes

qPCR was used to verify the expression status of selected genes identified from the RNASeq analysis. For these experiments, different animals from the same two cohorts as the RNASeq experiment were used. Four animals at M and four animals at AII, thus 8 total animals, were tested for righting and TWR, then sacrificed for RNA extraction as previously described for RNASeq. RNA was quantified on a Nanodrop (model ND-1000), and 100 ng of RNA was reverse transcribed to cDNA using the SuperScript III First-Strand Synthesis System with random hexamer primers (Invitrogen). The resulting cDNA was diluted 1:5 with nuclease-free H_2_O to provide the working concentration of cDNA.

Primers designed for each gene were 18–22 bp and generated target amplicons of 75–125 bp (See Additional file 1: Table S1 for primer sequences and amplicon lengths). To assess amplification of the desired target sequences, each set of primers was tested by PCR amplification of cDNA. PCR products were separated on a 1% agarose gel to confirm the appropriate size for each amplicon. These bands from the agarose gel were then cut out and the DNA purified using a Qiagen Gel Purification kit, and purified gel products were sequenced by Genewiz (South Plainfield, NJ) to confirm amplification of the desired target sequence.

qPCR reactions were carried out on a Stratagene Mx3005P with SYBR Green master mix under the following conditions: 95 °C for 10 min, followed by 40 cycles of 95 °C for 15 s, 58 °C for 30 s, 72 °C for 30 s. For each biological replicate two technical replicates were performed. All runs were normalized to GAPDH control and relative standard curves were used for analysis. Expression ratios were calculated for genes between M and AII using the Gene Expression’s C_T_ Difference formula [24], a modified version of the ΔΔC_T_ method [25]. This formula accounts for different efficiencies in each reaction by using calculating the efficiency of each qPCR reaction independently using Real-time PCR miner [26]. These efficiencies were then used to calculate relative transcription for each sample as previously described [24, 25].

## Results

### Behavioral changes in TWR and righting with age

**Fig. 1:**
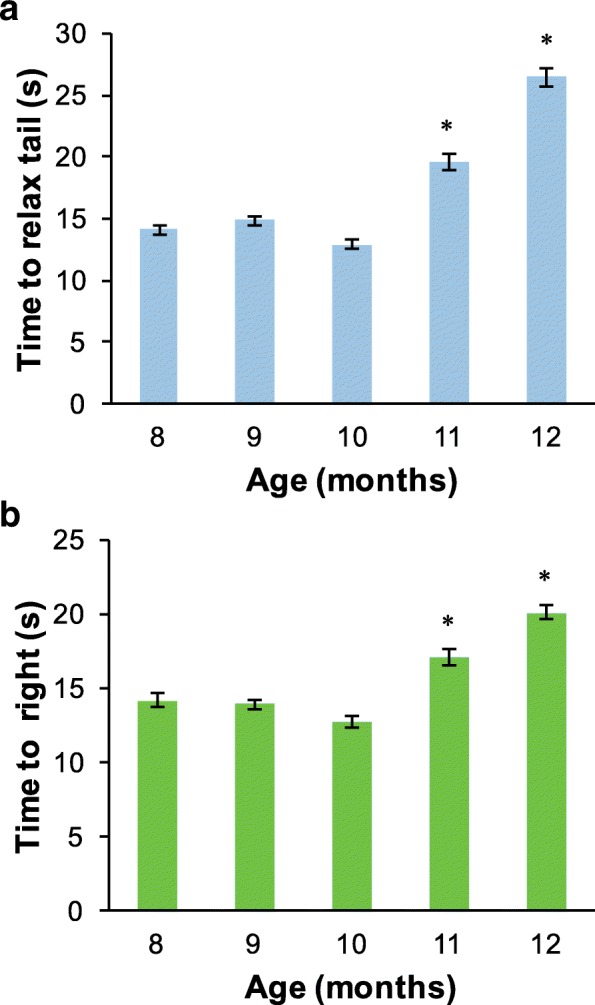
Behavioral assessments of aging in cohorts used for RNASeq. Time to complete TWR (**a**) and righting reflex (**b**) increased significantly with age. All data presented as mean ± SEM * denotes significant difference compared to other ages, p≤ 0.05, One-way ANOVA, Tukey’s post-hoc

No significant differences were observed in either TWR or righting between the two cohorts at any age (Student’s t-test, p≤0.05). Therefore, times were combined from the two cohorts for analysis of behavioral aging. TWR significantly increased with age in both cohorts (Figure 1a, *p* < 0.05). Time to right also increased significantly with age (Figure 1b, *p* < 0.05). Designation of the age 12 month animals as AII was consistent with the previously defined stages of aging in Aplysia [5], namely age > 11 months, TWR time > 20.1 s, and righting time > 18 s.

### Read quality assessment

After quality control filtering, a total of ~ 210 million reads were used for downstream analysis (an average of ~ 17.5 million reads/individual). Quality score boxplots of the reads showed that base quality scores were high for all 100 bp, indicating high quality sample preparation and sequencing (Additional file 1: Figure S1).

Genomic mapping of reads with STAR resulted in 50–70% of reads per individual uniquely mapped to the NCBI *Aplysia californica* Annotation Release 101 genome (assembly AplCal3.0). See Additional file 1: Table S2 for a summary of read depth and mapping for each library with the STAR.

### Gene expression changes in aged animals

**Fig. 2:**
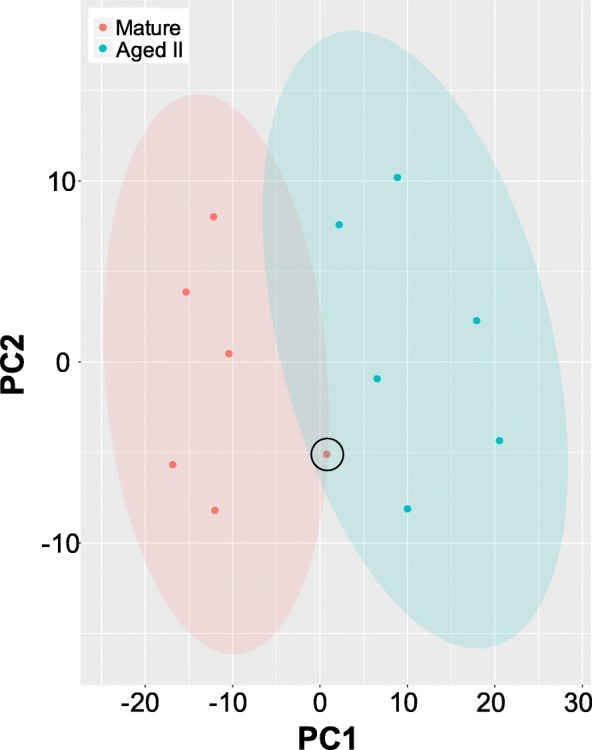
Principal component analysis (PCA) in M and AII PVC neurons. Gene expression profiles of M and AII PVC neurons plotted against the first two principal components, with clustering indicating expression profiles were most similar within each age group. One M animal exhibited an expression profile that did not clearly belong to either age group (circled). Light red and blue shading represent 95% confidence interval for M and AII, respectively

The correlation between the biological replicates within each age group was calculated with a PCA to determine if overall gene expression patterns differ between M and AII animals (Figure 2). The first two principal components accounted for 57% of the variability in the dataset. Most biological replicates clustered together, indicating that expression profiles were most similar between individuals belonging to the same age group. One exception was a single M animal that showed an intermediate expression profile that did not clearly belong to either M or AII (Figure 2, circled).

**Fig. 3:**
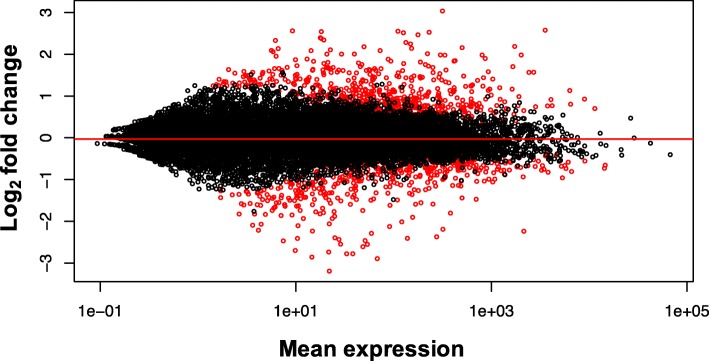
Scatterplot of mean expression for each gene and log-fold change. Points in red indicate significant DE at padj≤0.05. Negative log-fold changes along the y-axis corresponds to transcripts that showed lower expression in AII animals

**Fig. 4:**
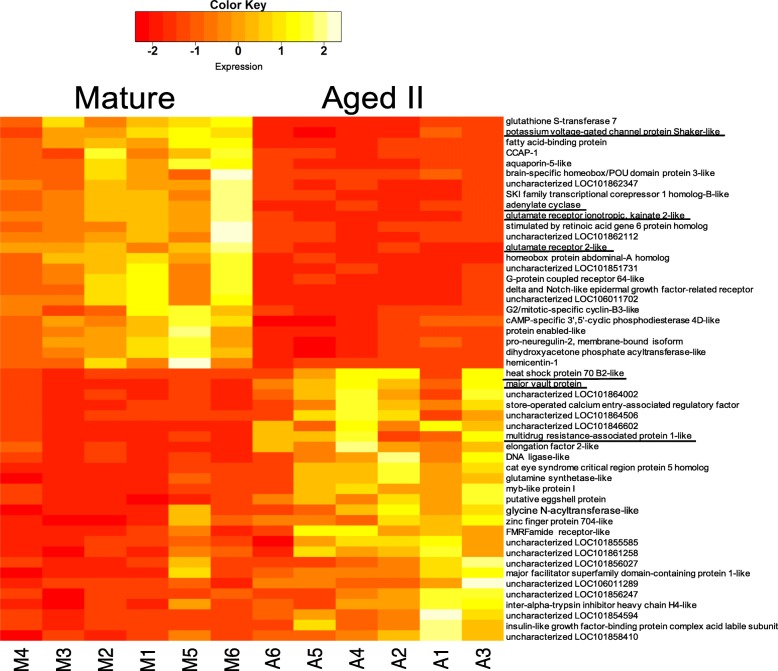
Heatmap of the top 50 most significantly DE genes. Genes that were upregulated with age include several stress related genes such as heat shock protein and major vault protein (underlined). Many transcripts related to nervous system function were downregulated in aged animals (underlined). Yellow indicates higher expression compared to the average across all replicates, and red indicates reduced expression

There were 1202 DE genes identified between M and AII sensory neurons at padj≤0.05 (full list of DE transcripts available in Additional file 2). Approximately as many genes were upregulated as were downregulated in aged PVC neurons (Figure 3). Increased expression in AII was observed for 655 of the 1202 DE genes (55.3%), and 537 genes (46.7%) had decreased expression with age. A heatmap showing the 50 genes with the most significant changes in expression (lowest *p*-value) can be found in Figure 4. Several of the genes in this list that exhibited decreased expression in AII animals play a role in neuronal excitability, including voltage-gated potassium channels, voltage-gated calcium channels, and ionotropic glutamate receptors (iGluR). Other genes with decreased expression are involved in induction of long-term memory, including adenylate cyclase (Figure 4) and the catalytic subunit of protein kinase A (PKA; Additional file 2). Several transcripts that were upregulated in AII play a role in cell protection and oxidative stress response, including heat shock protein, major vault protein, and multi-drug resistance protein.

DE was analyzed between the two cohorts, without accounting for age, to determine if batch effects occurred. We found only 25 DE transcripts between the two cohorts (padj≤0.05, data not shown), indicating that there were few differences in gene expression due to the biological variability of the two cohorts. None of these 25 genes were also DE in the aging analysis. Therefore, differences in expression in this study are likely attributed to transcriptional changes between M and AII.

### Changes in iGluR subunit associated with aging

**Fig. 5:**
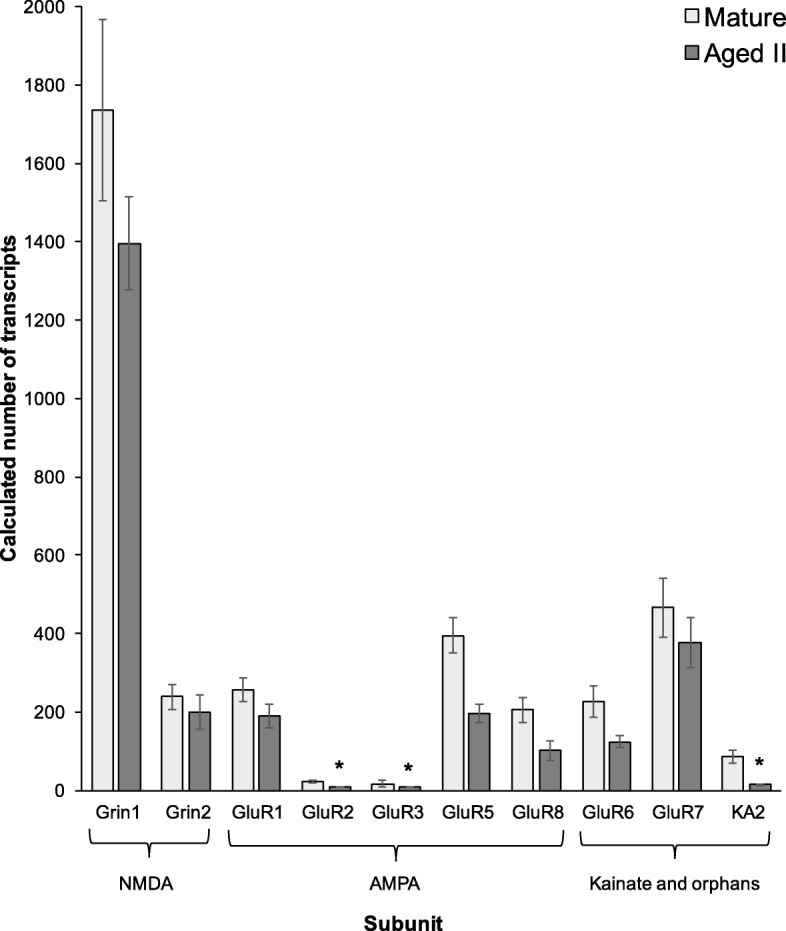
Expression of iGluR subunits in M and AII PVC neurons. Calculated number of iGluR transcripts in AII compared to M in PVC sensory neurons showed down regulation of some iGluR genes in AII. Data is expressed as mean ± SEM * denotes significantly decreased expression compared to M (padj≤0.05)

iGluR subunits were analyzed to test if reduced expression of specific iGluR coincided with previously observed declines in glutamatergic neurotransmission in AII PVC neurons. The NMDA receptor subunit Grin1accounted for the majority of expression and was not down regulated in AII, nor was Grin2 (Figure 5). GluR2 and GluR3, belonging to the AMPA subtype, and KA2, a kainate subunit [27], were significantly down regulated in AII. GluR4 and KA1 subunits were not analyzed due to a low number of counts for these two genes.

### Gene ontology analysis

DE genes were mapped to gene ontology pathways using Blast2GO to determine the biological processes and molecular functions that were most significantly affected during aging. BLAST hits matching human proteins were identified for 1054 of the 1202 DE *Aplysia* proteins (E-value<1E-3), of which 844 were unique human proteins (Additional file 2). Approximately 1000 gene ontology terms were found to be over-represented in the analysis (padj≤0.05), suggesting that many functions were significantly altered in AII animals (complete list of enriched terms available in Additional file 3).

Several of the most significantly affected molecular functions in PVC neurons are involved in ion channel function (voltage-gated ion channel activity, voltage-gated channel activity, ion channel binding, voltage-gated cation channel activity, potassium channel activity, cation channel activity, ion channel activity, and channel activity). Furthermore, when combining these categories, ~ 80% of DE ion channel associated genes showed reduced expression in aged animals (Additional file 4). This suggested that decreased ion channel function occurred in aged animals. Synaptic transmission was also a significantly enriched biological process, further emphasizing alterations in neuronal transmission in AII (Additional file 3).

Response to stress was also found to be an enriched biological process (Additional file 3). Approximately 70% of DE stress response genes showed increased expression in aged animals, consistent with findings of increased stress response with age in many other species [28].

### Canonical pathways altered in aging

**Fig. 6:**
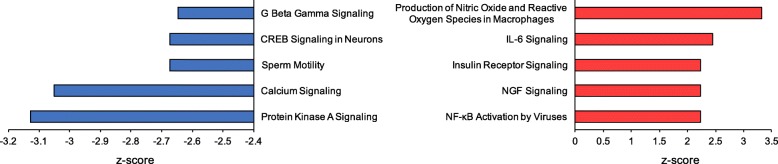
Canonical pathways predicted to have the largest changes in aging. Negative z-scores (blue bars) indicate pathways predicted to decline in function with age. Pathways predicted to be enhanced in aged animals (positive z-scores, red bars) include production of nitric oxide and ROS, associated with aging in many species

The 844 DE genes corresponding to unique human proteins in Blast2GO were analyzed in IPA to further explore canonical pathways that were up- or down regulated in aged PVC sensory neurons. Pathways with the five highest and five lowest z-scores can be found in Figure 6 (full list of pathways available in Additional file 1: Table S3).

**Fig. 7:**
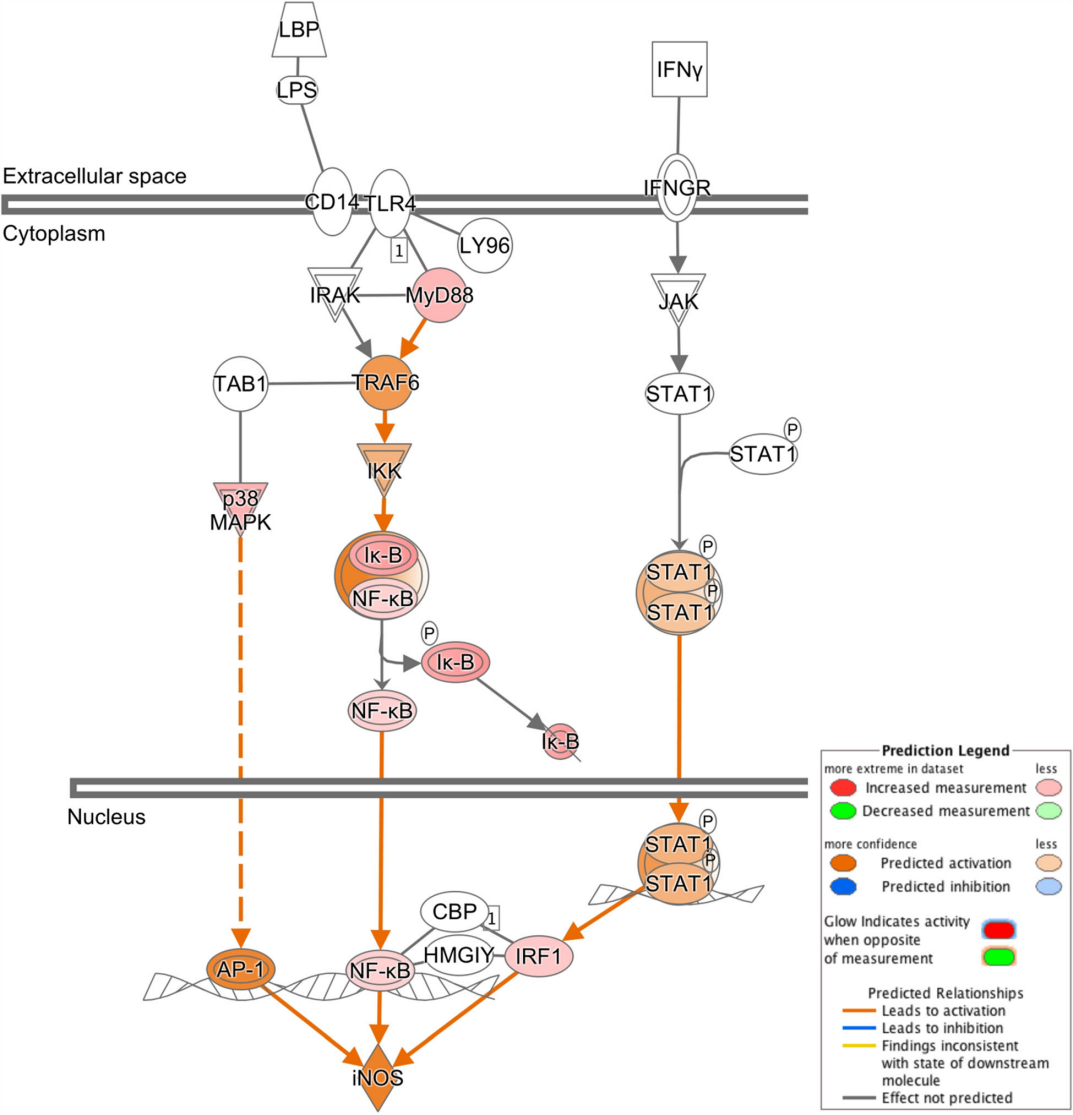
Activation of the iNOS pathway with age. Production of nitric oxide via iNOS was predicted to increase in aged neurons. Two transcriptional activators of iNOS, MAPK and NF-Κb, increased expression (padj≤0.05). A third, STAT1, was predicted by IPA to have increased expression. High concentrations of nitric oxide have been implicated in aging and may result in increased DNA damage, apoptosis, and neuronal death

Pathways with positive z-scores indicate increased activity in aged neurons, and included stress pathways such as production of nitric oxide and reactive oxygen species (z = 3.317, Figure 6) and the NRF2-mediated oxidative stress response pathway (z = 1.633, Additional file 1: Table S3, Additional file 3). Increased nitric oxide (NO) was predicted via the inducible nitric oxide synthase pathway (iNOS) due to increased expression of two transcriptional activators: nuclear factor kappa-light-chain-enhancer of activated B cells (NF-κB) and MAPK (Figure 7). IPA also predicted increased activation of another transcriptional regulator involved with NO production, STAT1. Nerve growth factor (NGF) signaling, important in maintenance, survival, and plasticity of neurons, also had increased activation in aged neurons (Figure 6).

**Fig. 8:**
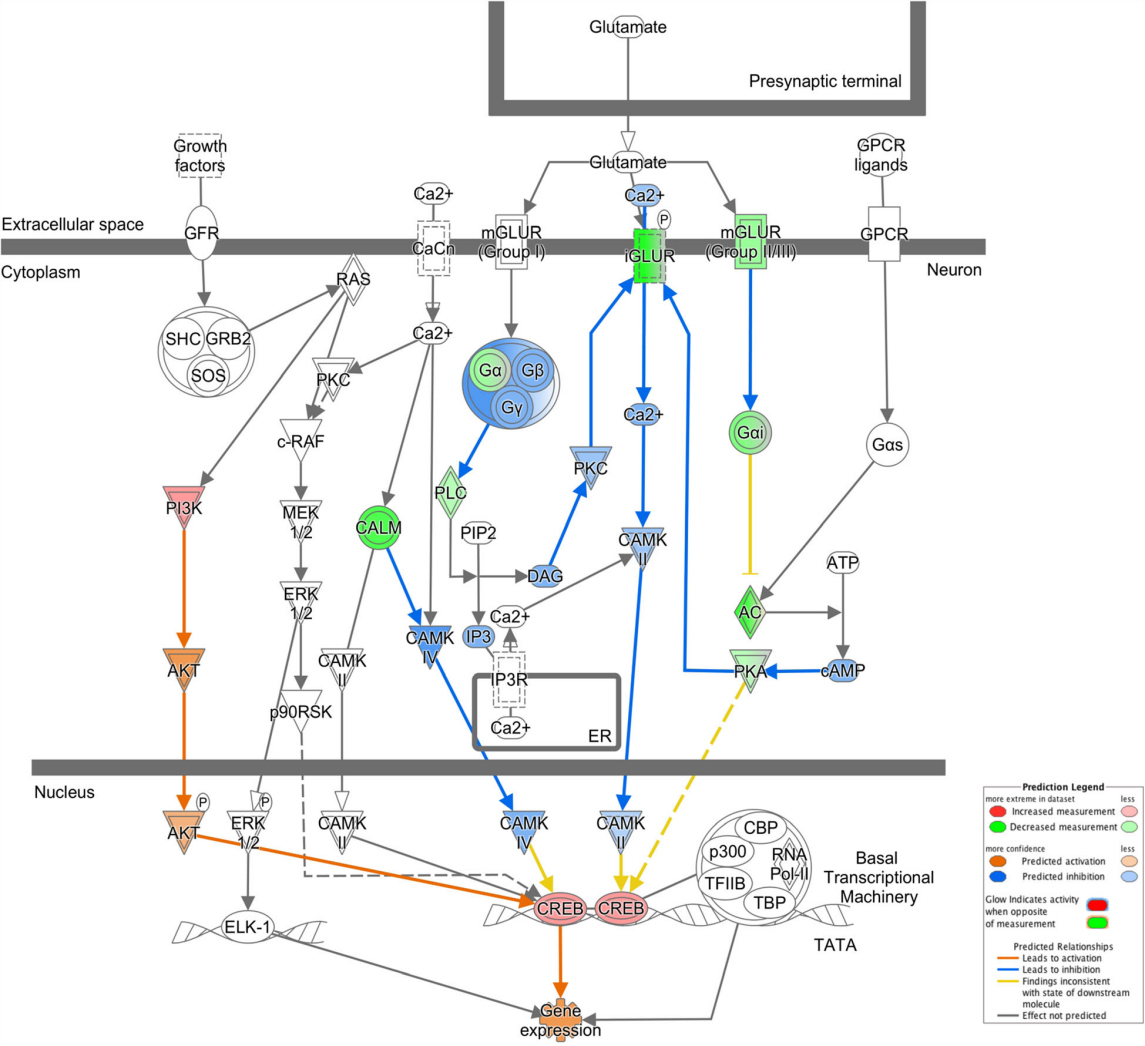
Alterations in CREB signaling. Many genes in the CREB signaling pathway showed altered expression with age, including PKA, an important second messenger in neurons necessary for activation of both CREB and iGluR. See Figure 5 for specific iGluR genes that were downregulated

PKA signaling and CREB signaling both showed negative z-scores, indicating reduced activity of these pathways with age (z = − 3.218, − 2.673, respectively; Figure 6). Visualization of the CREB signaling pathway shows predicted regulation of iGluRs by both PKC and PKA. Lowered levels of these regulators are predicted by IPA to reduce iGluR expression (Figure 8). This was consistent with observed values the experimental data between PKA and several iGluRs (GluR2, GluR3, and KA2). PKC was not found to be differentially expressed in the database.

The glutamate receptor signaling pathway was also significantly decreased in aged neurons (z = − 1.342). PKA is predicted to directly affect iGluR expression as well (Figure 8). Thus, reduced expression of PKA may play an important role in both reduced iGluR expression and CREB signaling in aged animals. CREB activation was predicted in this pathway based on observed up regulation of one component gene of the complex, CREB3-like 3, and the activation of PI3K, which may also stimulate CREB complex activation. Thus, we see an inconsistency where pathways involving iGluRs and intracellular calcium levels would be expected to reduce CREB complex expression while those driven by PI3K would predict elevated CREB levels. Actual levels of CREB1 and CREB2 were not significantly altered in aged neurons suggesting that no clear, dominant effect was seen on this pathway.

Analysis of gene regulator networks revealed that voltage-gated calcium and potassium channels, in addition to iGluRs, may also be downregulated in aged Aplysia neurons. A predicted decrease in PKC may have resulted in decreased expression measurements of some ion channels (see Additional file 1: Figure S2).

### qPCR verification of changes in gene expression observed by RNASeq

A subset of nine genes important to neuronal excitability in humans were quantified via qPCR at M and AII, using new RNA samples from the same two cohorts sampled for RNASeq. Six of the 9 genes selected for qPCR showed significant DE in RNASeq. These six genes coded for ion channel proteins: voltage-gated potassium channel Shaw (Shaw), voltage-gated potassium channel Shaker (Shaker), voltage-dependent calcium channel subunit alpha-2/delta-3, which regulates kinetics and current amplitude through voltage-gated Ca^2+^ channels, and sodium channel alpha-subunit SCAP1. CREB3 was chosen for its role in cell communication and calcium ion transport. CCAAT/enhancer-binding protein (C/EBP) is a transcription factor important for transcriptional regulation of many genes.

**Fig. 9:**
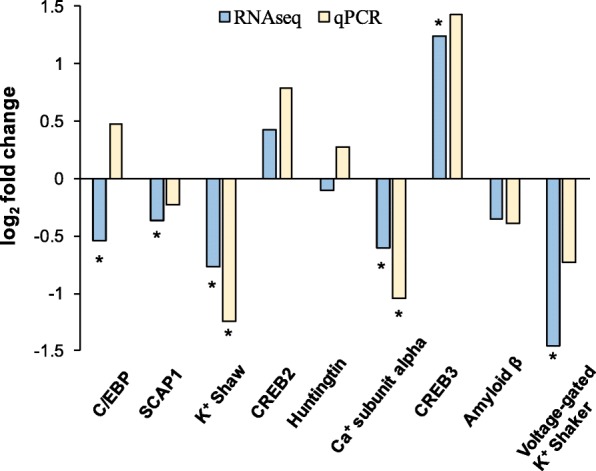
Comparison of DE genes in RNASeq and qPCR analyses. Negative log-fold change indicates reduced expression in AII animals. K^+^ channel Shaw and voltage-dependent Ca^2+^ channel subunit alpha-2/delta-3 were the only two genes significantly DE in both analyses (qPCR *n* = 4, RNASeq *n* = 6). *denotes significantly different expression compared to M (p≤0.05, Student’s t-test)

Three other genes tested via qPCR, CREB2, amyloid protein β, and huntingtin were not found to be DE in RNASeq, but were included for qPCR as genes previously tested in Aplysia nervous tissue. CREB2, amyloid protein β, and huntingtin were not found to be different by qPCR. On the other hand, two ion channel genes, Shaw and voltage-dependent calcium channel subunit alpha-2/delta-3, had significantly different expression in aged animals measured by both RNASeq and qPCR (Figure 9, p≤0.05). There were no significant changes in expression for the remaining 7 genes via qPCR.

## Discussion

Aplysia is a well-used model of aging in the nervous system due to a relatively compact life span and well-mapped neural circuits [8, 29–31]. Many Aplysia genes have homologs in higher vertebrates, making it a relevant model for age-related changes in the brain [32]. Furthermore, Aplysia contains homologs for many genes that are relevant to neurological diseases associated with age in humans [33]. We found that > 85% of the DE transcripts aligned to human transcripts in BLAST searches (E-value≤1E-3). This allowed for mapping most DE genes to the much better-characterized human gene ontologies for pathway enrichment, and allowed for predictions of pathways that may be important in aging vertebrates.

Previous transcriptome studies on aging in Aplysia and other model organisms have found genes to be both up- and downregulated with age [12, 33, 34]. While downregulation is to be expected as organ systems slow and fail, upregulation is observed in stress response [29], longevity enhancement, energy maintenance [35], and genes related to preserving physiological function, such as neurotransmission [36]. Thus, it was unsurprising that aging of PVC neurons exhibited bidirectional changes in gene expression, with ~ 55% of DE genes displaying increased expression in AII. This is approximately the same percentage of genes up regulated in the R15 bursting neuron of the Aplysia abdominal ganglion during aging [12].

The AII animals used for RNASeq in this experiment showed significant behavioral deficits in TWR compared to M animals. Reduced excitability of PVC sensory neurons to both tail taps and intracellular current injection has been proposed to significantly contribute to reduced TWR in AII Aplysia [5]. GO analysis showed DE of genes and gene ontology categories that would coincide with the reduced excitation observed in aged PVC neurons compared to mature. Ion channel associated GO categories were among the most significantly enriched categories in Blast2GO. When ion channel categories were combined, 65 out of the 425 identified genes exhibited altered expression in AII, with 82% of the 65 genes downregulated, suggesting reduced function with age. Ten DE ion channel associated genes encode for voltage-gated K^+^ and Ca^2+^ channel proteins, all of which were downregulated in AII. The loss of functional gated K^+^ and Ca^2+^ channels may be a crucial molecular mechanism underlying reduced excitability previously demonstrated in AII PVC neurons, leading to subsequent declines in TWR during aging. Reduced excitability during aging has been described in many other parts of the Aplysia nervous system as well [5, 8, 11, 15], and is correlated with memory declines in the aging mammalian hippocampus and [2, 37–40]. Declines in neuronal excitability appear to be widespread in the aging nervous systems of both mammals and Aplysia.

In addition to reduced excitability, previous studies showed AII PVC neurons exhibited reduced density of L-Glu-induced excitatory currents [5]. Three iGluR subunits, GluR2, GluR3, belonging to the AMPA subtype, and KA2, a kainate subunit, showed significantly reduced expression in AII PVC neurons compared to M via RNASeq. This suggests reduced L-Glu current amplitude in AII PVC sensory neurons [5] may be due to decreased expression of iGluR. However, the differentially expressed subunits were not highly expressed. The most highly expressed subunits (Grin1, GluR5, and GluR7) were not DE. Therefore, reduced L-Glu current amplitude in AII PVC sensory neurons may be due to aging changes at the protein level, for example, in PKA or PKC-mediated modulation of iGluR currents.

IPA identified aging-related decreases in both CREB signaling and PKA signaling, which play large roles in learning and memory and maintenance of iGluR signaling via receptor phosphorylation [41], respectively. The adenylate cyclase gene also had decreased expression. Its activation of PKA phosphorylates CREB, to increase synaptic strength by inducing the transcription of many genes necessary for facilitation. All would likely be impaired by the reduced expression of these genes observed in aging, and are consistent with impaired learning and memory, as well as neurophysiology, noted in aged Aplysia [42]. Direct injection of PKA into AII PVC neurons rescued facilitation, suggesting that processes upstream of PKA activation, such as adenylate cyclase, may be compromised during aging [43].

Decreased expression of genes involved in synaptic plasticity during aging have been implicated in the declines in memory tasks described in many species [44–46]. Reduced expression of genes involved in learning and memory systems appears to be a ubiquitous effect of aging in the nervous system of animals via reduced transcription of components necessary for induction of facilitation.

In contrast with down regulated genes involved in neuronal excitation, numerous genes involved in stress responses are up regulated in aged Aplysia. Stress response is an evolutionarily conserved feature of aging, occurring in countless species from roundworms to humans [28], and has previously been noted in Aplysia abdominal neurons [12, 33]. Accumulation of reactive oxygen species (ROS) in aging results in oxidative damage that induces DNA mutations detrimental to cell survival, including altering the structure and function of many macromolecules [47] and repressed transcription of neuronal genes [48]. Oxidation of ion channels due to ROS is wide spread during aging, and can modulate functional changes in voltage-gated Na^+^, K^+^, and Ca^2+^ channels [49, 50]. Nrf2-mediated oxidative stress response and production of ROS were identified as pathways with increased activity in aged sensory neurons. Nrf2 is translocated from the cytoplasm to the nucleus to bind DNA promoters and increase transcription of stress response genes [51]. Increased levels of inducible nitric oxide synthase (iNOS) also were predicted by IPA, another inducer of stress response that is implicated to promote brain aging via such diverse actions such as inhibition of cell proliferation, DNA damage, apoptosis, aggravation of age-related neurodegenerative diseases, neurotoxicity, and impaired cognition [52–56].

Additional genes with increased expression in AII PVC neurons that have been implicated in stress, chemoprotection, and cell survival include heat shock proteins, major vault protein, and multi-drug resistance protein. Four heat shock proteins were upregulated in AII PVC neurons, including hsp70, which refolds or degrades severely damaged proteins caused by ROS [57–60]. Other genes implicated in chemoprotection and cell survival with increased expression included major vault protein, suggested to play a role in cell signaling and prevention of stress-induced apoptosis whose expression was increased in aged humans [61], and multi-drug resistance protein, with a role in responding to ROS. Increased expression of these genes in AII PVC neurons may be a compensatory mechanism to maintain proper homeostasis by preventing ROS and other oxidative damage accumulated during aging.

Another important neuronal pathway with increased activity during aging was nerve growth factor signaling. NGF signaling includes processes necessary to maintain proper neuronal function and maintenance of neural connections, further suggesting altered neural function occurs in aged sensory neurons of Aplysia. Altered expression of NGF pathways has also previously been linked to Alzheimer’s and other neural diseases [62, 63].

Amyloid β protein and huntingtin, genes implicated in other neurological diseases and reduced neuronal function in aged humans [32], were tested for DE with qPCR. Amyloid β is the major component of amyloid plaques associated with neuronal impairments related to Alzheimer’s disease [64], and was one of the most highly expressed transcripts in this study. Huntingtin is a protein critical for induction of learning in Aplysia [65], and expression of mutant huntingtin induced neurological deficits in Huntington’s disease mouse models [66, 67]. Neither gene was DE, suggesting that aging and impaired physiology of PVC neurons in Aplysia may be due to normal aging.

## Conclusions

This study examined whole-transcriptome changes to analyze Aplysia sensory neuron aging for the first time. Aging in PVC neurons coincided with decreased expression and altered pathways for many plasma membrane proteins important to excitability, such as voltage-gated K^+^ channels, voltage-gated Ca^2+^ channels, and iGluR. Altered ion channel expression with age may underlie reduced sensory neuron performance observed in AII Aplysia. Upregulation of stress response genes and decreased expression of genes important for long term memory are evolutionarily conserved changes with age that were also observed.

## Additional files

Additional file 1: **Table S1.** Primer sequences and amplicon lengths for qPCR. **Table S2.** Read depth and mapping statistics using fastxtoolkit and STAR. **Table S3.** List of canonical pathways identified in IPA. **Figure S1.** Representative quality score boxplot for reads after quality filtering. **Figure S2.** Visualization of regulatory network involving ion channel genes from IPA. (DOCX 5196 kb)

Additional file 2: Excel file containing all differentially expressed genes identified by STAR. (XLSX 58 kb)

Additional file 3: Excel file with a complete list of GO ontologies enriched in Blast2GO analysis. This file contains *p*-values and sequences identified for each ontology as well. (XLSX 3392 kb)

Additional file 4: Excel file listing all ion channel genes identified in Blast2GO. (XLSX 175 kb)

## Abbreviations

AII: Aged II (age 12 months)
C/EBP: CCAAT/enhancer-binding protein
CNS: Central nervous system
DE: Differential expression
GO: Gene ontology
iGluR: Ionotropic glutamate receptor
iNOS: Inducible nitric oxide synthase
IPA: Ingenuity Pathway Analysis
M: Mature (age 8 months)
NF-κb: Nuclear factor kappa-light-chain-enhancer of activated B cells
NGF: Nerve growth factor
NO: Nitric oxide
padj: Adjusted *p*-value
PCA: Principal component analysis
PKA: Protein kinase A
PVC: Pleural ventral caudal
ROS: Reactive oxygen species
TWR: Tail-withdrawal reflex
