# Supplementary material for: Whole-transcriptome changes in gene expression accompany aging of sensory neurons in Aplysia californica
Source: BMC Genomics. 2018 Jul 11;19:529. doi: 10.1186/s12864-018-4909-1 (PMC6042401; doi:10.1186/s12864-018-4909-1)
Supplement: Supplementary file 1 — Table S1. Primer sequences and amplicon lengths for qPCR. Table S2. Read depth and mapping statistics using fastxtoolkit and STAR. Table S3. List of canonical pathways identified in IPA. Figure S1. Representative quality score boxplot for reads after quality filtering. Figure S2. Visualization of regulatory network involving ion channel genes from IPA. (DOCX 5196 kb) [file 12864_2018_4909_MOESM1_ESM.docx]

| Page | Figure |
| --- | --- |
| 2 | **Table S1. Primer sequences for qPCR** |
| 3 | **Table S2. Read depth and mapping statistics using fastxtoolkit and STAR** |
| 4 | **Table S3. Canonical pathways identified in IPA** |
| 12 | **Fig. S1. Quality score boxplot of reads after quality filtering** |
| 13 | **Fig. S2. Predicted regulatory network involving ion channel genes** |

**Table S1. Primer sequences for qPCR.** Forward and reverse primers used for qPCR validation of 9 genes from RNASeq.

| Gene | Primer Sequences for qPCR (5’-3’) | | Amplicon length (bp) |
| --- | --- | --- | --- |
| CCAAT/enhancer-binding protein zeta-like | FR | ATCAACTCAGCCGACGTCTC  GGGACTGCTTCTTCGTCTTGA | 114 |
| sodium channel alpha-subunit SCAP1 | F  R | ATCGGCATGCAGCTCTACTC  ACCCAATCGTTCCACTCGTC | 104 |
| potassium voltage-gated channel protein Shaw-like | F  R | TGGACATAAAGGCGTGTTGC  GACTGTCATCTGTTCCCGCT | 104 |
| CREB2 | F  R | TTGTTGGGTGGCATGGAACT  AGTTCTTCAGCACCGCCAAT | 107 |
| Huntingtin | F  R | TGGACACTCAGACCACCAGT  CTCTAATAACGCTGCACGGA | 76 |
| voltage-dependent calcium channel subunit alpha-2/delta-3 | F  R | ACTTATGAGGGCCTGGGTCT  ACGTCCGTTCCTACAACACC | 122 |
| CREB3B | F  R | TGCCAACGAACTTACCCCTC  CCTTTTGCGGCTCTCTTTCG | 101 |
| amyloid beta A4 | F  R | ATCAGAATAGACGACGCGGG  CTGCCTCTCTCGTAGTCGTG | 101 |
| potassium voltage-gated channel protein Shaker-like | F  R | GCCACTGGGGAAGCTCATAG  CCTCTTCCAGTCTTGTGCGA | 120 |

**Table S2. Read depth and mapping statistics using fastxtoolkit and STAR.** Reads are separated into paired and unpaired, as that lost their mate during quality control steps are counted as unpaired. The total number of transcripts counted by htSEQ includes only reads that mapped that were mapped to exons by STAR.

| Sample |  | Total Number of reads after Trimming | % reads mapped to genome by STAR | Total # transcripts counted by htSEQ |
| --- | --- | --- | --- | --- |
| Mature1 | paired | 19,608,062 | 66.76% | 3,090,792 |
|  | unpaired | 1,037,824 | 79.24% |  |
| Mature2 | paired | 14,783,156 | 70.54% | 2,482,607 |
|  | unpaired | 956,055 | 84.06% |  |
| Mature3 | paired | 13,576256 | 72.74% | 2,582,177 |
|  | unpaired | 918,426 | 81.13% |  |
| Mature4 | paired | 14,511,446 | 61.82% | 2,187,782 |
|  | unpaired | 627,032 | 60.07% |  |
| Mature5 | paired | 25,116,528 | 72.62% | 4,549,233 |
|  | unpaired | 1,502,596 | 75.38% |  |
| Mature6 | paired | 28,406,435 | 50.64% | 3,108,397 |
|  | unpaired | 1,886,646 | 77.29% |  |
| Aged1 | paired | 18,443,688 | 59.36% | 2,664,852 |
|  | unpaired | 1,113,916 | 77.29% |  |
| Aged2 | paired | 18,051,002 | 63.18% | 2,525,682 |
|  | unpaired | 960,838 | 57.74% |  |
| Aged3 | paired | 17,130,158 | 60.77% | 2,768,254 |
|  | unpaired | 1,157,305 | 75.96% |  |
| Aged4 | paired | 15,336,780 | 58.36% | 1,911,932 |
|  | unpaired | 710,853 | 45.04% |  |
| Aged5 | paired | 14,527,360 | 60.41% | 1,464,547 |
|  | unpaired | 656,870 | 56.38% |  |
| Aged6 | paired | 10,089,056 | 52.91% | 1,525,506 |
|  | unpaired | 832,514 | 57.39% |  |

**Table S3. Canonical pathways identified in IPA.** Differentially expressed genes with human homologs were analyzed in IPA for pathways affected by aging. The ratio column indicates the percentage of genes in the pathway that were DE. Z-score take into account direction of regulation for genes in the pathway to make predictions about downstream processes. A negative score is a prediction that the given pathway will be downregulated, a positive score predicts increased pathway activity.

| **Canonical Pathway** | **-log(p-value)** | **Ratio** | **z-score** |
| --- | --- | --- | --- |
| Protein Kinase A Signaling | 3.91 | 0.074 | -3.128 |
| Calcium Signaling | 5.37 | 0.112 | -3.051 |
| Sperm Motility | 5.14 | 0.128 | -2.673 |
| CREB Signaling in Neurons | 2.69 | 0.0815 | -2.673 |
| G Beta Gamma Signaling | 1.47 | 0.0795 | -2.646 |
| eNOS Signaling | 2.97 | 0.0903 | -2.496 |
| Melatonin Signaling | 3.11 | 0.127 | -2.333 |
| Corticotropin Releasing Hormone Signaling | 4.5 | 0.126 | -2.309 |
| cAMP-mediated signaling | 4.43 | 0.0942 | -2.236 |
| fMLP Signaling in Neutrophils | 2.47 | 0.0909 | -2.111 |
| Relaxin Signaling | 6.46 | 0.132 | -1.941 |
| Synaptic Long Term Depression | 2.74 | 0.089 | -1.941 |
| Gαs Signaling | 2.32 | 0.0917 | -1.897 |
| Neuropathic Pain Signaling In Dorsal Horn Neurons | 2.19 | 0.0877 | -1.897 |
| Synaptic Long Term Potentiation | 2.03 | 0.0833 | -1.897 |
| Chemokine Signaling | 1.93 | 0.0986 | -1.89 |
| Cardiac β-adrenergic Signaling | 3.51 | 0.102 | -1.732 |
| Dopamine-DARPP32 Feedback in cAMP Signaling | 4.28 | 0.105 | -1.698 |
| Dopamine Receptor Signaling | 1.75 | 0.0909 | -1.633 |
| PPAR Signaling | 0.976 | 0.0645 | -1.633 |
| PPARα/RXRα Activation | 4.28 | 0.101 | -1.604 |
| Antioxidant Action of Vitamin C | 2.01 | 0.0874 | -1.414 |
| GNRH Signaling | 3.24 | 0.101 | -1.387 |
| TWEAK Signaling | 2.22 | 0.147 | -1.342 |
| Glutamate Receptor Signaling | 1.85 | 0.105 | -1.342 |
| Androgen Signaling | 1.4 | 0.0721 | -1.342 |
| Regulation of Actin-based Motility by Rho | 0.676 | 0.0549 | -1.342 |
| CDK5 Signaling | 2.63 | 0.101 | -1.265 |
| α-Adrenergic Signaling | 4.33 | 0.138 | -1.155 |
| LPS/IL-1 Mediated Inhibition of RXR Function | 1.95 | 0.0676 | -1.134 |
| Melanocyte Development and Pigmentation Signaling | 1.32 | 0.0737 | -1.134 |
| RhoA Signaling | 1.2 | 0.0656 | -1.134 |
| Role of NFAT in Regulation of the Immune Response | 0.947 | 0.0541 | -1.134 |
| Wnt/β-catenin Signaling | 0.625 | 0.0473 | -1.134 |
| Ephrin Receptor Signaling | 0.583 | 0.046 | -1.134 |
| Wnt/Ca+ pathway | 0.864 | 0.0702 | -1 |
| Actin Cytoskeleton Signaling | 0.76 | 0.0482 | -1 |
| Ceramide Signaling | 0.39 | 0.043 | -1 |
| Fcγ Receptor-mediated Phagocytosis in Macrophages and Monocytes | 0.39 | 0.043 | -1 |
| Sumoylation Pathway | 0.366 | 0.0417 | -1 |
| 14-3-3-mediated Signaling | 0.323 | 0.0385 | -1 |
| Glioma Signaling | 0.271 | 0.0364 | -1 |
| Nitric Oxide Signaling in the Cardiovascular System | 3.24 | 0.106 | -0.905 |
| AMPK Signaling | 1.81 | 0.0688 | -0.905 |
| Induction of Apoptosis by HIV1 | 1.75 | 0.1 | -0.816 |
| Role of PI3K/AKT Signaling in the Pathogenesis of Influenza | 1.31 | 0.0789 | -0.816 |
| BMP signaling pathway | 1.31 | 0.0789 | -0.816 |
| CD28 Signaling in T Helper Cells | 1.06 | 0.0611 | -0.816 |
| Apoptosis Signaling | 1.91 | 0.0899 | -0.707 |
| CXCR4 Signaling | 0.91 | 0.0545 | -0.707 |
| Thrombin Signaling | 1.59 | 0.064 | -0.577 |
| Colorectal Cancer Metastasis Signaling | 1.29 | 0.0567 | -0.535 |
| Cardiac Hypertrophy Signaling | 2.43 | 0.0723 | -0.5 |
| Activation of IRF by Cytosolic Pattern Recognition Receptors | 1.19 | 0.0806 | -0.447 |
| IGF-1 Signaling | 1.11 | 0.066 | -0.447 |
| LXR/RXR Activation | 0.608 | 0.0496 | -0.447 |
| Cdc42 Signaling | 0.278 | 0.0359 | -0.447 |
| Gαq Signaling | 0.969 | 0.0562 | -0.378 |
| Sphingosine-1-phosphate Signaling | 0.86 | 0.0569 | -0.378 |
| Renin-Angiotensin Signaling | 1.61 | 0.075 | -0.333 |
| Phospholipase C Signaling | 0.871 | 0.05 | -0.333 |
| Integrin Signaling | 0.451 | 0.0411 | -0.333 |
| P2Y Purigenic Receptor Signaling Pathway | 2.19 | 0.0833 | -0.302 |
| IL-1 Signaling | 4.8 | 0.143 | -0.277 |
| Death Receptor Signaling | 4.08 | 0.13 | 0 |
| April Mediated Signaling | 1.37 | 0.105 | 0 |
| Agrin Interactions at Neuromuscular Junction | 1.03 | 0.0725 | 0 |
| Signaling by Rho Family GTPases | 0.809 | 0.0486 | 0 |
| p70S6K Signaling | 0.762 | 0.0534 | 0 |
| iCOS-iCOSL Signaling in T Helper Cells | 0.598 | 0.0492 | 0 |
| PI3K Signaling in B Lymphocytes | 0.541 | 0.0469 | 0 |
| Endothelin-1 Signaling | 1.85 | 0.0695 | 0.277 |
| Gαi Signaling | 1.61 | 0.075 | 0.333 |
| ERK/MAPK Signaling | 0.802 | 0.0503 | 0.333 |
| TNFR1 Signaling | 1.56 | 0.102 | 0.447 |
| IL-17A Signaling in Airway Cells | 0.884 | 0.0649 | 0.447 |
| CD40 Signaling | 0.867 | 0.0641 | 0.447 |
| Huntington's Disease Signaling | 0.656 | 0.0456 | 0.447 |
| Rac Signaling | 0.651 | 0.0513 | 0.447 |
| RhoGDI Signaling | 1.09 | 0.0578 | 0.707 |
| TNFR2 Signaling | 1.76 | 0.138 | 1 |
| ILK Signaling | 0.611 | 0.0459 | 1 |
| Mouse Embryonic Stem Cell Pluripotency | 0.295 | 0.0377 | 1 |
| mTOR Signaling | 0.265 | 0.0352 | 1 |
| MIF-mediated Glucocorticoid Regulation | 2.27 | 0.152 | 1.342 |
| MIF Regulation of Innate Immunity | 1.87 | 0.122 | 1.342 |
| Paxillin Signaling | 0.696 | 0.0531 | 1.342 |
| Tec Kinase Signaling | 0.419 | 0.0412 | 1.342 |
| RANK Signaling in Osteoclasts | 2.56 | 0.099 | 1.414 |
| UVA-Induced MAPK Signaling | 1.58 | 0.0784 | 1.414 |
| NF-κB Signaling | 1.29 | 0.0611 | 1.508 |
| NRF2-mediated Oxidative Stress Response | 2.49 | 0.0777 | 1.633 |
| Retinoic acid Mediated Apoptosis Signaling | 2.29 | 0.115 | 1.633 |
| Toll-like Receptor Signaling | 1.84 | 0.0946 | 1.633 |
| TREM1 Signaling | 1.33 | 0.08 | 1.633 |
| Acute Phase Response Signaling | 0.625 | 0.0473 | 1.633 |
| B Cell Receptor Signaling | 0.704 | 0.0486 | 1.667 |
| Dendritic Cell Maturation | 1.46 | 0.0632 | 1.732 |
| PKCθ Signaling in T Lymphocytes | 0.75 | 0.053 | 1.89 |
| CD27 Signaling in Lymphocytes | 2.04 | 0.115 | 2 |
| PEDF Signaling | 1.14 | 0.0714 | 2 |
| Type II Diabetes Mellitus Signaling | 0.809 | 0.0551 | 2 |
| STAT3 Pathway | 0.603 | 0.0548 | 2 |
| Type I Diabetes Mellitus Signaling | 1.42 | 0.0727 | 2.121 |
| PI3K/AKT Signaling | 1.17 | 0.0645 | 2.121 |
| Role of Pattern Recognition Receptors in Recognition of Bacteria and Viruses | 0.974 | 0.0584 | 2.121 |
| iNOS Signaling | 3.12 | 0.159 | 2.236 |
| LPS-stimulated MAPK Signaling | 0.743 | 0.0581 | 2.236 |
| NF-κB Activation by Viruses | 0.743 | 0.0581 | 2.236 |
| NGF Signaling | 0.413 | 0.0427 | 2.236 |
| Insulin Receptor Signaling | 0.262 | 0.0355 | 2.236 |
| IL-6 Signaling | 0.55 | 0.0472 | 2.449 |
| Production of Nitric Oxide and Reactive Oxygen Species in Macrophages | 1.12 | 0.057 | 3.317 |
| PXR/RXR Activation | 6.51 | 0.2 | N/A |
| Cellular Effects of Sildenafil (Viagra) | 5.55 | 0.131 | N/A |
| Glycolysis I | 4.65 | 0.269 | N/A |
| Gap Junction Signaling | 4.55 | 0.106 | N/A |
| Hepatic Cholestasis | 4.38 | 0.107 | N/A |
| Role of PKR in Interferon Induction and Antiviral Response | 4.22 | 0.2 | N/A |
| G-Protein Coupled Receptor Signaling | 4.06 | 0.0846 | N/A |
| Unfolded protein response | 4.03 | 0.167 | N/A |
| TCA Cycle II (Eukaryotic) | 3.97 | 0.261 | N/A |
| Gluconeogenesis I | 3.65 | 0.231 | N/A |
| Netrin Signaling | 3.45 | 0.179 | N/A |
| Role of NFAT in Cardiac Hypertrophy | 3.38 | 0.0885 | N/A |
| Xenobiotic Metabolism Signaling | 3.31 | 0.0767 | N/A |
| Breast Cancer Regulation by Stathmin1 | 3.11 | 0.0837 | N/A |
| GPCR-Mediated Integration of Enteroendocrine Signaling Exemplified by an L Cell | 3.07 | 0.125 | N/A |
| Mitochondrial Dysfunction | 3.01 | 0.0877 | N/A |
| L-cysteine Degradation III | 2.92 | 1 | N/A |
| Acetyl-CoA Biosynthesis I (Pyruvate Dehydrogenase Complex) | 2.88 | 0.429 | N/A |
| Aspartate Degradation II | 2.88 | 0.429 | N/A |
| tRNA Charging | 2.67 | 0.154 | N/A |
| Superpathway of Geranylgeranyldiphosphate Biosynthesis I (via Mevalonate) | 2.62 | 0.235 | N/A |
| GPCR-Mediated Nutrient Sensing in Enteroendocrine Cells | 2.55 | 0.106 | N/A |
| Glutamate Degradation II | 2.45 | 0.667 | N/A |
| Aspartate Biosynthesis | 2.45 | 0.667 | N/A |
| Sertoli Cell-Sertoli Cell Junction Signaling | 2.41 | 0.0787 | N/A |
| Gustation Pathway | 2.39 | 0.0887 | N/A |
| autophagy | 2.33 | 0.117 | N/A |
| Endoplasmic Reticulum Stress Pathway | 2.26 | 0.19 | N/A |
| L-cysteine Degradation I | 2.16 | 0.5 | N/A |
| Acetate Conversion to Acetyl-CoA | 2.16 | 0.5 | N/A |
| Acyl-CoA Hydrolysis | 2.14 | 0.25 | N/A |
| Mevalonate Pathway I | 2.03 | 0.231 | N/A |
| Guanosine Nucleotides Degradation III | 2.03 | 0.231 | N/A |
| Leptin Signaling in Obesity | 2.02 | 0.0941 | N/A |
| tRNA Splicing | 1.96 | 0.128 | N/A |
| Isoleucine Degradation I | 1.94 | 0.214 | N/A |
| Urate Biosynthesis/Inosine 5'-phosphate Degradation | 1.94 | 0.214 | N/A |
| Colanic Acid Building Blocks Biosynthesis | 1.94 | 0.214 | N/A |
| Caveolar-mediated Endocytosis Signaling | 1.93 | 0.0986 | N/A |
| Hepatic Fibrosis / Hepatic Stellate Cell Activation | 1.92 | 0.071 | N/A |
| Molecular Mechanisms of Cancer | 1.92 | 0.0588 | N/A |
| Stearate Biosynthesis I (Animals) | 1.87 | 0.122 | N/A |
| Chondroitin Sulfate Degradation (Metazoa) | 1.85 | 0.2 | N/A |
| Glutaryl-CoA Degradation | 1.85 | 0.2 | N/A |
| Superpathway of Cholesterol Biosynthesis | 1.81 | 0.143 | N/A |
| Glycine Cleavage Complex | 1.78 | 0.333 | N/A |
| Granzyme B Signaling | 1.77 | 0.188 | N/A |
| Dermatan Sulfate Degradation (Metazoa) | 1.77 | 0.188 | N/A |
| Parkinson's Signaling | 1.77 | 0.188 | N/A |
| Adenosine Nucleotides Degradation II | 1.7 | 0.176 | N/A |
| Clathrin-mediated Endocytosis Signaling | 1.68 | 0.066 | N/A |
| 4-1BB Signaling in T Lymphocytes | 1.66 | 0.129 | N/A |
| Valine Degradation I | 1.63 | 0.167 | N/A |
| Hypoxia Signaling in the Cardiovascular System | 1.6 | 0.0923 | N/A |
| Virus Entry via Endocytic Pathways | 1.58 | 0.0784 | N/A |
| Small Cell Lung Cancer Signaling | 1.57 | 0.0833 | N/A |
| GABA Receptor Signaling | 1.54 | 0.0896 | N/A |
| Inositol Pyrophosphates Biosynthesis | 1.53 | 0.25 | N/A |
| Purine Nucleotides Degradation II (Aerobic) | 1.51 | 0.15 | N/A |
| Aldosterone Signaling in Epithelial Cells | 1.51 | 0.0663 | N/A |
| Amyloid Processing | 1.5 | 0.098 | N/A |
| IL-12 Signaling and Production in Macrophages | 1.5 | 0.0685 | N/A |
| Epithelial Adherens Junction Signaling | 1.5 | 0.0685 | N/A |
| Tight Junction Signaling | 1.49 | 0.0659 | N/A |
| IL-17A Signaling in Fibroblasts | 1.49 | 0.114 | N/A |
| Glutamine Biosynthesis I | 1.46 | 1 | N/A |
| Asparagine Biosynthesis I | 1.46 | 1 | N/A |
| Maturity Onset Diabetes of Young (MODY) Signaling | 1.45 | 0.143 | N/A |
| Oxidative Phosphorylation | 1.44 | 0.0734 | N/A |
| Triacylglycerol Degradation | 1.44 | 0.0943 | N/A |
| Phototransduction Pathway | 1.44 | 0.0943 | N/A |
| Amyotrophic Lateral Sclerosis Signaling | 1.4 | 0.0721 | N/A |
| Notch Signaling | 1.37 | 0.105 | N/A |
| Ketogenesis | 1.34 | 0.2 | N/A |
| Tumoricidal Function of Hepatic Natural Killer Cells | 1.31 | 0.125 | N/A |
| Tryptophan Degradation III (Eukaryotic) | 1.31 | 0.125 | N/A |
| B Cell Activating Factor Signaling | 1.3 | 0.1 | N/A |
| Dolichyl-diphosphooligosaccharide Biosynthesis | 1.27 | 0.182 | N/A |
| Ethanol Degradation IV | 1.26 | 0.12 | N/A |
| Lipid Antigen Presentation by CD1 | 1.22 | 0.115 | N/A |
| NAD Salvage Pathway II | 1.22 | 0.115 | N/A |
| Role of RIG1-like Receptors in Antiviral Innate Immunity | 1.21 | 0.093 | N/A |
| Serotonin Receptor Signaling | 1.21 | 0.093 | N/A |
| Role of Osteoblasts, Osteoclasts and Chondrocytes in Rheumatoid Arthritis | 1.2 | 0.056 | N/A |
| Protein Ubiquitination Pathway | 1.2 | 0.0549 | N/A |
| β-alanine Degradation I | 1.16 | 0.5 | N/A |
| Spermidine Biosynthesis I | 1.16 | 0.5 | N/A |
| Sulfate Activation for Sulfonation | 1.16 | 0.5 | N/A |
| Axonal Guidance Signaling | 1.16 | 0.049 | N/A |
| RAR Activation | 1.16 | 0.0579 | N/A |
| Oleate Biosynthesis II (Animals) | 1.13 | 0.154 | N/A |
| NAD Phosphorylation and Dephosphorylation | 1.13 | 0.154 | N/A |
| Role of Macrophages, Fibroblasts and Endothelial Cells in Rheumatoid Arthritis | 1.12 | 0.0518 | N/A |
| Atherosclerosis Signaling | 1.12 | 0.063 | N/A |
| Androgen Biosynthesis | 1.08 | 0.143 | N/A |
| Phenylalanine Degradation IV (Mammalian, via Side Chain) | 1.08 | 0.143 | N/A |
| Lymphotoxin β Receptor Signaling | 1.08 | 0.0746 | N/A |
| Role of IL-17A in Arthritis | 1.08 | 0.0746 | N/A |
| CCR3 Signaling in Eosinophils | 1.07 | 0.0615 | N/A |
| Glutathione-mediated Detoxification | 1.07 | 0.1 | N/A |
| Sonic Hedgehog Signaling | 1.07 | 0.1 | N/A |
| Remodeling of Epithelial Adherens Junctions | 1.03 | 0.0725 | N/A |
| p53 Signaling | 1.03 | 0.0631 | N/A |
| Telomere Extension by Telomerase | 1.03 | 0.133 | N/A |
| nNOS Signaling in Skeletal Muscle Cells | 1.03 | 0.133 | N/A |
| Regulation of eIF4 and p70S6K Signaling | 1.01 | 0.0573 | N/A |
| Superpathway of Methionine Degradation | 1 | 0.0938 | N/A |
| Diphthamide Biosynthesis | 0.996 | 0.333 | N/A |
| NADH Repair | 0.996 | 0.333 | N/A |
| Inosine-5'-phosphate Biosynthesis II | 0.996 | 0.333 | N/A |
| Hypusine Biosynthesis | 0.996 | 0.333 | N/A |
| Thiosulfate Disproportionation III (Rhodanese) | 0.996 | 0.333 | N/A |
| 4-aminobutyrate Degradation I | 0.996 | 0.333 | N/A |
| Role of MAPK Signaling in the Pathogenesis of Influenza | 0.975 | 0.0694 | N/A |
| Docosahexaenoic Acid (DHA) Signaling | 0.971 | 0.0769 | N/A |
| Inhibition of Angiogenesis by TSP1 | 0.944 | 0.0882 | N/A |
| Salvage Pathways of Pyrimidine Ribonucleotides | 0.943 | 0.0632 | N/A |
| Aryl Hydrocarbon Receptor Signaling | 0.934 | 0.0571 | N/A |
| γ-linolenate Biosynthesis II (Animals) | 0.933 | 0.118 | N/A |
| Interferon Signaling | 0.889 | 0.0833 | N/A |
| Uracil Degradation II (Reductive) | 0.879 | 0.25 | N/A |
| Pentose Phosphate Pathway (Oxidative Branch) | 0.879 | 0.25 | N/A |
| Branched-chain α-keto acid Dehydrogenase Complex | 0.879 | 0.25 | N/A |
| Thymine Degradation | 0.879 | 0.25 | N/A |
| Geranylgeranyldiphosphate Biosynthesis | 0.879 | 0.25 | N/A |
| Proline Biosynthesis I | 0.879 | 0.25 | N/A |
| Glycerol-3-phosphate Shuttle | 0.879 | 0.25 | N/A |
| Ethanol Degradation II | 0.863 | 0.0811 | N/A |
| GADD45 Signaling | 0.852 | 0.105 | N/A |
| Regulation of IL-2 Expression in Activated and Anergic T Lymphocytes | 0.851 | 0.0633 | N/A |
| ATM Signaling | 0.834 | 0.0625 | N/A |
| Nur77 Signaling in T Lymphocytes | 0.825 | 0.0678 | N/A |
| MSP-RON Signaling Pathway | 0.825 | 0.0678 | N/A |
| Estrogen Biosynthesis | 0.814 | 0.0769 | N/A |
| Inhibition of Matrix Metalloproteases | 0.814 | 0.0769 | N/A |
| Neuroprotective Role of THOP1 in Alzheimer's Disease | 0.79 | 0.075 | N/A |
| Retinol Biosynthesis | 0.79 | 0.075 | N/A |
| 2-ketoglutarate Dehydrogenase Complex | 0.789 | 0.2 | N/A |
| 2-oxobutanoate Degradation I | 0.789 | 0.2 | N/A |
| Trans, trans-farnesyl Diphosphate Biosynthesis | 0.789 | 0.2 | N/A |
| Galactose Degradation I (Leloir Pathway) | 0.789 | 0.2 | N/A |
| dTMP De Novo Biosynthesis | 0.789 | 0.2 | N/A |
| Glutamate Degradation III (via 4-aminobutyrate) | 0.789 | 0.2 | N/A |
| Oxidative Ethanol Degradation III | 0.783 | 0.0952 | N/A |
| Phospholipases | 0.771 | 0.0645 | N/A |
| T Cell Receptor Signaling | 0.744 | 0.055 | N/A |
| HIPPO signaling | 0.729 | 0.0575 | N/A |
| Pyrimidine Deoxyribonucleotides De Novo Biosynthesis I | 0.721 | 0.087 | N/A |
| Pyridoxal 5'-phosphate Salvage Pathway | 0.72 | 0.0615 | N/A |
| Proline Biosynthesis II (from Arginine) | 0.718 | 0.167 | N/A |
| Arginine Degradation VI (Arginase 2 Pathway) | 0.718 | 0.167 | N/A |
| Thioredoxin Pathway | 0.718 | 0.167 | N/A |
| Glycerol Degradation I | 0.718 | 0.167 | N/A |
| Glycogen Biosynthesis II (from UDP-D-Glucose) | 0.718 | 0.167 | N/A |
| Rapoport-Luebering Glycolytic Shunt | 0.718 | 0.167 | N/A |
| GDP-mannose Biosynthesis | 0.718 | 0.167 | N/A |
| Crosstalk between Dendritic Cells and Natural Killer Cells | 0.702 | 0.0562 | N/A |
| Eicosanoid Signaling | 0.689 | 0.0597 | N/A |
| IL-9 Signaling | 0.686 | 0.0667 | N/A |
| IL-10 Signaling | 0.673 | 0.0588 | N/A |
| IL-17A Signaling in Gastric Cells | 0.666 | 0.08 | N/A |
| CCR5 Signaling in Macrophages | 0.659 | 0.058 | N/A |
| NAD Biosynthesis from 2-amino-3-carboxymuconate Semialdehyde | 0.658 | 0.143 | N/A |
| Glycoaminoglycan-protein Linkage Region Biosynthesis | 0.658 | 0.143 | N/A |
| nNOS Signaling in Neurons | 0.649 | 0.0638 | N/A |
| Myc Mediated Apoptosis Signaling | 0.644 | 0.0571 | N/A |
| Prostate Cancer Signaling | 0.638 | 0.0532 | N/A |
| Ovarian Cancer Signaling | 0.625 | 0.0486 | N/A |
| Phagosome Maturation | 0.625 | 0.0486 | N/A |
| IL-15 Production | 0.616 | 0.0741 | N/A |
| Acetone Degradation I (to Methylglyoxal) | 0.616 | 0.0741 | N/A |
| Basal Cell Carcinoma Signaling | 0.616 | 0.0556 | N/A |
| Cell Cycle: G2/M DNA Damage Checkpoint Regulation | 0.614 | 0.0612 | N/A |
| Superoxide Radicals Degradation | 0.607 | 0.125 | N/A |
| Salvage Pathways of Pyrimidine Deoxyribonucleotides | 0.607 | 0.125 | N/A |
| Phagosome Formation | 0.598 | 0.0492 | N/A |
| Germ Cell-Sertoli Cell Junction Signaling | 0.591 | 0.0462 | N/A |
| FAK Signaling | 0.581 | 0.0505 | N/A |
| Intrinsic Prothrombin Activation Pathway | 0.572 | 0.069 | N/A |
| Role of Wnt/GSK-3β Signaling in the Pathogenesis of Influenza | 0.564 | 0.0526 | N/A |
| Prostanoid Biosynthesis | 0.564 | 0.111 | N/A |
| Sucrose Degradation V (Mammalian) | 0.564 | 0.111 | N/A |
| Ketolysis | 0.564 | 0.111 | N/A |
| Angiopoietin Signaling | 0.552 | 0.0519 | N/A |
| Estrogen-Dependent Breast Cancer Signaling | 0.552 | 0.0519 | N/A |
| VDR/RXR Activation | 0.54 | 0.0513 | N/A |
| Erythropoietin Signaling | 0.528 | 0.0506 | N/A |
| Embryonic Stem Cell Differentiation into Cardiac Lineages | 0.525 | 0.1 | N/A |
| Glucose and Glucose-1-phosphate Degradation | 0.525 | 0.1 | N/A |
| Pentose Phosphate Pathway | 0.525 | 0.1 | N/A |
| Glucocorticoid Receptor Signaling | 0.523 | 0.0418 | N/A |
| G Protein Signaling Mediated by Tubby | 0.513 | 0.0625 | N/A |
| Fatty Acid β-oxidation I | 0.513 | 0.0625 | N/A |
| Actin Nucleation by ARP-WASP Complex | 0.509 | 0.0536 | N/A |
| Circadian Rhythm Signaling | 0.495 | 0.0606 | N/A |
| Purine Nucleotides De Novo Biosynthesis II | 0.491 | 0.0909 | N/A |
| UDP-N-acetyl-D-galactosamine Biosynthesis II | 0.491 | 0.0909 | N/A |
| Heparan Sulfate Biosynthesis | 0.484 | 0.0482 | N/A |
| Agranulocyte Adhesion and Diapedesis | 0.471 | 0.0423 | N/A |
| Regulation of the Epithelial-Mesenchymal Transition Pathway | 0.471 | 0.0423 | N/A |
| Cell Cycle Regulation by BTG Family Proteins | 0.461 | 0.0571 | N/A |
| Cleavage and Polyadenylation of Pre-mRNA | 0.46 | 0.0833 | N/A |
| Glycogen Degradation II | 0.46 | 0.0833 | N/A |
| BER pathway | 0.46 | 0.0833 | N/A |
| Assembly of RNA Polymerase III Complex | 0.433 | 0.0769 | N/A |
| PCP pathway | 0.424 | 0.0476 | N/A |
| DNA Double-Strand Break Repair by Non-Homologous End Joining | 0.407 | 0.0714 | N/A |
| Glycogen Degradation III | 0.407 | 0.0714 | N/A |
| Mitotic Roles of Polo-Like Kinase | 0.392 | 0.0455 | N/A |
| Transcriptional Regulatory Network in Embryonic Stem Cells | 0.389 | 0.05 | N/A |
| NAD biosynthesis II (from tryptophan) | 0.385 | 0.0667 | N/A |
| Vitamin-C Transport | 0.385 | 0.0667 | N/A |
| Mechanisms of Viral Exit from Host Cells | 0.376 | 0.0488 | N/A |
| Role of NANOG in Mammalian Embryonic Stem Cell Pluripotency | 0.376 | 0.041 | N/A |
| γ-glutamyl Cycle | 0.364 | 0.0625 | N/A |
| Methylglyoxal Degradation III | 0.364 | 0.0625 | N/A |
| Pyrimidine Ribonucleotides Interconversion | 0.352 | 0.0465 | N/A |
| FXR/RXR Activation | 0.349 | 0.0397 | N/A |
| CTLA4 Signaling in Cytotoxic T Lymphocytes | 0.343 | 0.0404 | N/A |
| Pyrimidine Ribonucleotides De Novo Biosynthesis | 0.33 | 0.0444 | N/A |
| PAK Signaling | 0.328 | 0.0396 | N/A |
| Ephrin B Signaling | 0.328 | 0.0411 | N/A |
| Gα12/13 Signaling | 0.323 | 0.0385 | N/A |
| Role of Oct4 in Mammalian Embryonic Stem Cell Pluripotency | 0.319 | 0.0435 | N/A |
| Triacylglycerol Biosynthesis | 0.319 | 0.0435 | N/A |
| VEGF Signaling | 0.315 | 0.0388 | N/A |
| Antiproliferative Role of Somatostatin Receptor 2 | 0.312 | 0.04 | N/A |
| Histamine Degradation | 0.31 | 0.0526 | N/A |
| IL-15 Signaling | 0.304 | 0.0395 | N/A |
| Heparan Sulfate Biosynthesis (Late Stages) | 0.304 | 0.0395 | N/A |
| Adipogenesis pathway | 0.299 | 0.0373 | N/A |
| EIF2 Signaling | 0.296 | 0.0362 | N/A |
| Cancer Drug Resistance By Drug Efflux | 0.29 | 0.0408 | N/A |
| Inflammasome pathway | 0.28 | 0.0476 | N/A |
| Macropinocytosis Signaling | 0.268 | 0.037 | N/A |
| Role of Lipids/Lipid Rafts in the Pathogenesis of Influenza | 0.266 | 0.0455 | N/A |
| Telomerase Signaling | 0.265 | 0.036 | N/A |
| Hereditary Breast Cancer Signaling | 0.257 | 0.0352 | N/A |
| Prolactin Signaling | 0.255 | 0.0361 | N/A |
| JAK/Stat Signaling | 0.255 | 0.0361 | N/A |
| Human Embryonic Stem Cell Pluripotency | 0.252 | 0.035 | N/A |
| IL-17 Signaling | 0.242 | 0.0353 | N/A |
| IL-22 Signaling | 0.242 | 0.0417 | N/A |
| Role of JAK1, JAK2 and TYK2 in Interferon Signaling | 0.242 | 0.0417 | N/A |
| Glutathione Redox Reactions I | 0.242 | 0.0417 | N/A |
| CDP-diacylglycerol Biosynthesis I | 0.242 | 0.0417 | N/A |
| Role of JAK family kinases in IL-6-type Cytokine Signaling | 0.231 | 0.04 | N/A |
| Bupropion Degradation | 0.231 | 0.04 | N/A |
| Phosphatidylglycerol Biosynthesis II (Non-plastidic) | 0.22 | 0.0385 | N/A |
| D-myo-inositol (1,4,5)-Trisphosphate Biosynthesis | 0.21 | 0.037 | N/A |


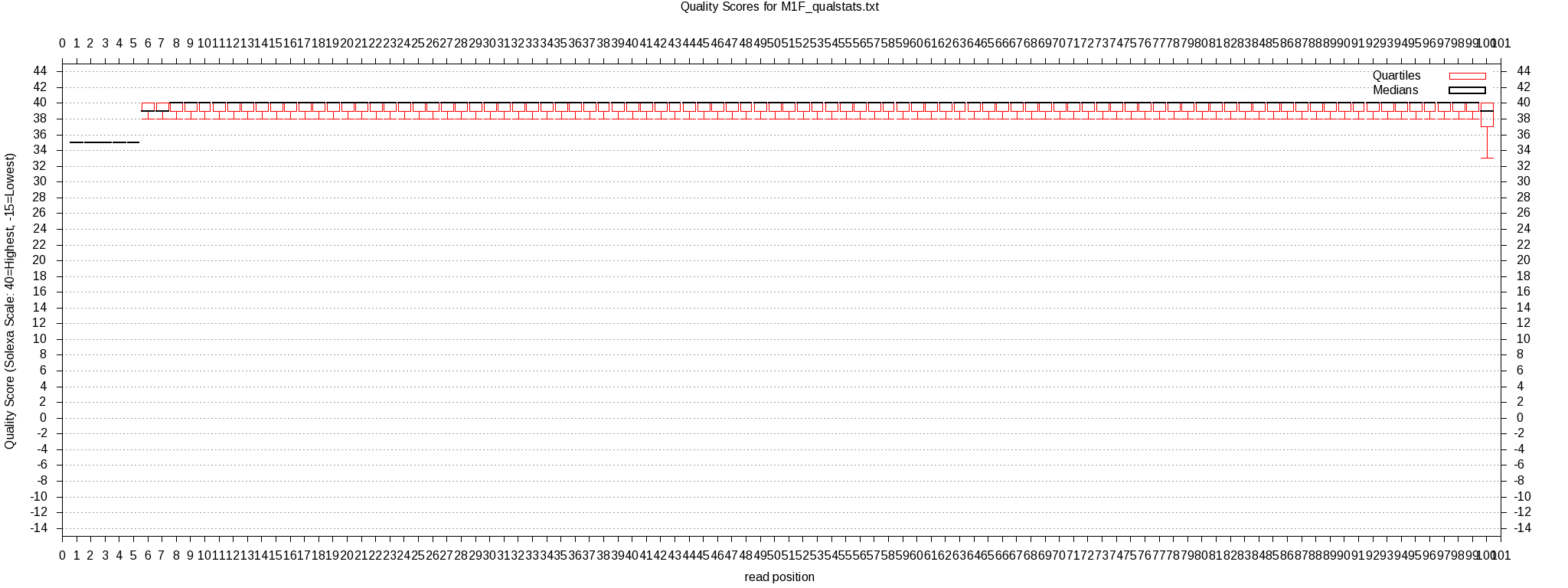


0

10

20

50

40

30

60

70

80

90

100

Read position

Quality score

**Fig. S1. Quality score boxplot of reads after quality filtering.** Representative boxplot for one RNASeq sample shows that high quality reads were obtained for all 100 base pairs. All other samples showed nearly identical boxplots.


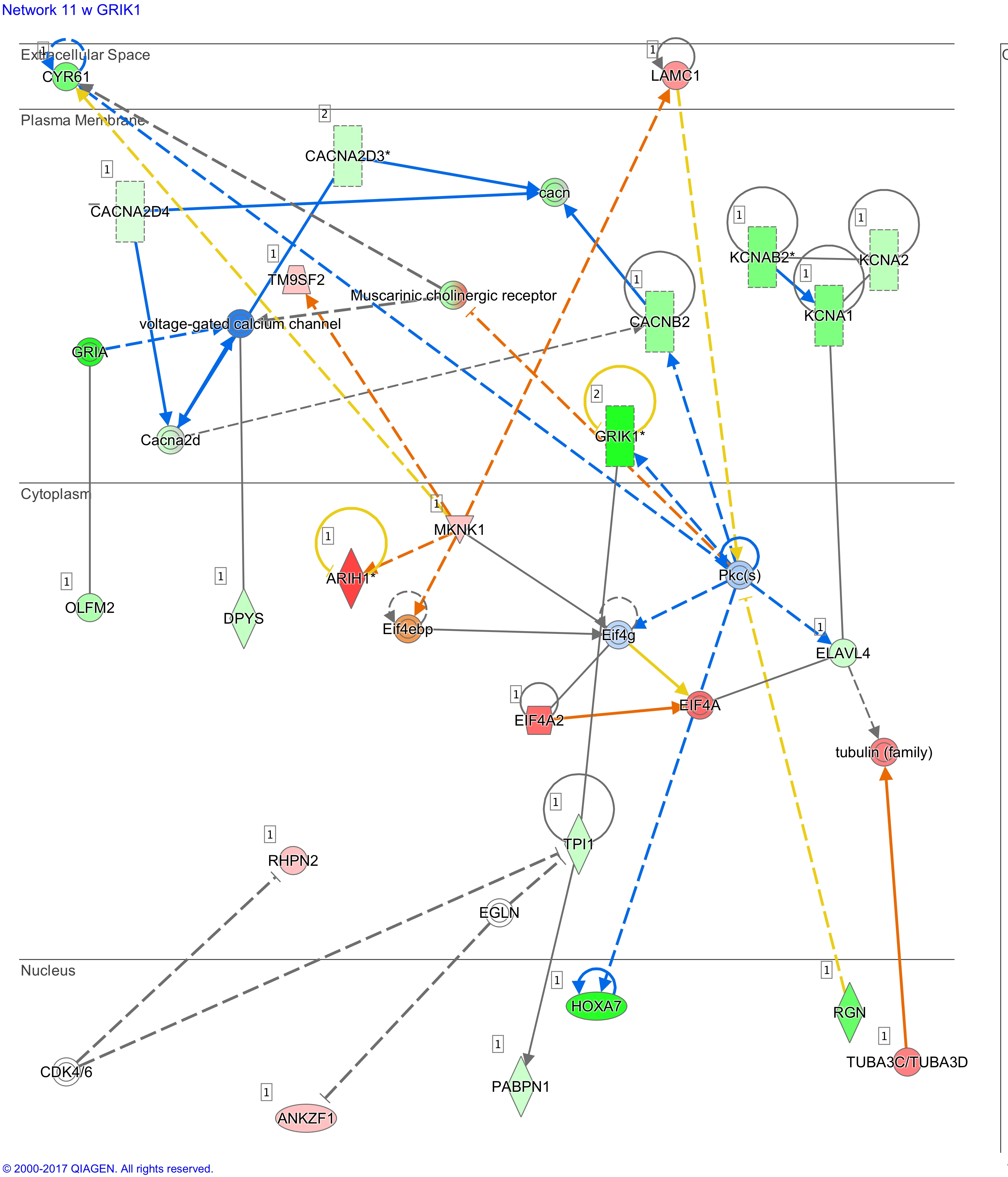

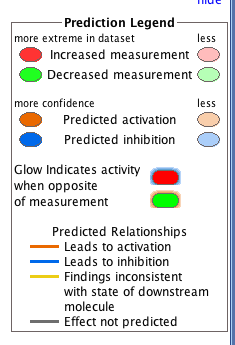


**Fig. S2. Predicted regulatory network involving ion channel genes**. This regulatory network shows observed changes in expression levels in PVC neurons in AII Aplysia and changes predicted by IPA (Network 11).
